# General Practitioners' Experiences During the First Phase of the COVID-19 Pandemic in Italy: A Critical Incident Technique Study

**DOI:** 10.3389/fpubh.2021.623904

**Published:** 2021-02-03

**Authors:** Peter Konstantin Kurotschka, Alice Serafini, Michela Demontis, Arianna Serafini, Alessandro Mereu, Maria Francesca Moro, Mauro Giovanni Carta, Luca Ghirotto

**Affiliations:** ^1^Department of Medical Sciences and Public Health, Faculty of Medicine and Surgery, University of Cagliari, Cagliari, Italy; ^2^Azienda Unità Sanitaria Locale di Modena, Modena, Italy; ^3^Regional Health Trust of Sardinia, Cagliari, Italy; ^4^Buontempi, Ferraresi & Lucchi Law Firm, Modena, Italy; ^5^Azienda Unità Sanitaria Locale Toscana Centro, Sesto Fiorentino, Firenze, Italy; ^6^Mailman School of Public Health, Columbia University, New York City, NY, United States; ^7^Qualitative Research Unit, Azienda Unità Sanitaria Locale – IRCCS di Reggio Emilia, Reggio Emilia, Italy

**Keywords:** pandemic, public health, doctor-patient relationship, health emergency, qualitative study, Italy, COVID−19, general practice

## Abstract

Since February 2020, when coronavirus disease began to spread in Italy, general practitioners (GPs) were called to manage a growing number of health situations. The challenges experienced by Italian GPs remained unrevealed. This study aimed at exploring Italian GPs' care experiences and practices associated with critical incidents during the first wave of the pandemic. A qualitative study design involving the critical incident technique through an online survey was applied. Sociodemographic data and open-ended responses were collected. While participants' characteristics were analyzed through descriptive statistics, qualitative data were thematically analyzed employing the framework method. 149 GPs responded to the survey and 99 participants completed the survey (dropout rate = 33%). Eight themes emerged indicating factors related to the organization of the healthcare system and factors related to the clinical management of patients, that were perceived as impacting on the GPs' care provision. The analysis revealed difficulties in communicating with other local services. This, together with the lack of coordination among services, was reported as a major challenge. Primary care was perceived as having been undervalued and criticalities in the organization of GP courses, led in a bureaucratic fashion, posed at risk some trainees to be infected. The digital technologies adopted for remote patient consultations were seen as useful tools for daily practice helping the GPs to stay emotionally connected with their patients. Besides, the improvement in the GP–patient relationship in terms of solidarity between patients and doctors and compliance to rules, had a positive impact. Moreover, many respondents addressed the importance of professional collaboration and teamwork, in terms of both support in practical issues (to find PPE, diagnostics and guidelines) and emotional support. At the same time, the lack of resources (e.g., PPE, swabs) and of specific guidelines and protocols impacted on the care provision. Our findings suggest that GPs in Italy are at risk of being left behind within the epidemic management. Communication and coordination among services are essential and should be substantially improved, and primary care research should be initiated to collect the context-specific evidence necessary to enhance the system's preparedness to public health emergencies and the quality of primary care services.

## Introduction

Italy was the first European country affected by the coronavirus disease (COVID-19) pandemic. Since the first case of COVID-19 was identified in Codogno, in the northern region of Lombardy, on February 20, 2020, severe acute respiratory syndrome coronavirus-2 (SARS-CoV-2) rapidly spread, mainly in northern Italy, partially sparing the southern regions of the country. To date, in Italy there have been more than 2 millions of confirmed cases, 71,620 deaths, and 23,571 people are currently hospitalized (1). Strict nationwide lockdown measures were adopted on March 9 and 11, 2020 (2, 3). During the lockdown, the Italian healthcare services were strongly challenged, especially regarding their capacity to deliver appropriate care to both COVID-19 patients and other patients. Outpatient secondary care services were closed to the public all over the country and planned patient consultations for non-life-threatening conditions were suspended. In this context, general practitioners (GPs), including out-of-hours doctors and doctors at prisons and nursing homes, were called to manage a growing number of health situations while reorganizing their services and altering how they provided care. Many GPs rapidly switched to remote consultations, though local, regional, and national evidence-based guidelines on COVID-19 management were lacking at that time. Services and care provision reorganization were left to the capacities of the individual GPs.

Rapidly moving to the frontline of COVID-19 management was demanding and put GPs in an unprecedented situation. Understanding the demands and challenges faced by frontline healthcare professionals and how they adjusted their efforts during the COVID-19 outbreak is essential (4). According to the World Health Organization (WHO), primary care services, in emergencies, should promote not only effective emergency responses but also a prepared system that can recover from emergencies (5). Primary care has been fundamental both for providing essential routine health services and for identifying/managing suspected COVID-19 patients (5–7).

Given the central role of primary care services during emergencies, we conducted a study collecting information on the experiences of Italian GPs during the COVID-19 pandemic. This study aimed at exploring Italian GPs' care experiences and practices associated with critical incidents during the first wave of the pandemic. In particular, this article reports on a qualitative analysis of “free text” (open-ended) survey data related to critical incidents experienced by Italian GPs. Findings reported in this article provide an insight on the GPs' experiences of positive as well as negative events arising from the crisis and on how GPs adapted to the changes of their activities. Obtaining a better understanding of the real difficulties and challenges faced by GPs could help to prevent them in the future. The research question which drove the study was: “what did help or hinder Italian GPs' activity during the first wave of the pandemic?”

## Materials and Methods

We used a qualitative study design involving the critical incident technique (CIT), as it was particularly consistent with the study aim (8, 9). The CIT is a qualitative methodology (rather than only a method) that involves a flexible set of principles (10). It does not focus on opinions but analyses the context of events. The critical incidents (CIs) (11) in this study refer to situations perceived as relevant by GPs dealing with the COVID-19 pandemic. The concept of “technique” in the CIT entails critical reflection focused on analyzing the human behaviors and contextual factors underlying the phenomena in question (12, 13).

As described by Viergever (10) CIT has commonly five steps: (1) description of the aim of the activity to deepen; (2) specifications of the nature of critical incidents to report and participants' characteristics; (3) data collection in line with the research question; (4) data analysis which includes pooling critical incidents into themes or areas; (5) interpretation and results' report.

As to the aim of the activity and the nature of related CIs (steps 1 and 2) the researchers planned to include a broad interest on care provision and clinical practice during the pandemic (especially referring to March and April 2020) of Italian GPs. In this context, reporting CIs was used to understand experienced obstacles and proposed solutions to the faced practical problems. Researchers decided to let the participants free to report any significant or important event (see Table 1) applying a broad-ranging version of CIT.

**Table 1:** Open-ended questions.

1.	In this period of emergency caused by SARS-CoV-2, thinking about your recent clinical practice, could you tell us one or more experiences that involved you personally and that surprised you positively?
2.	In this period of emergency caused by SARS-CoV-2, thinking about your recent clinical practice, could you tell us one or more experience that involved you personally and that surprised you negatively?
3.	Could you please tell us what you would change so that the facts you describe do not happen again in the future?

### Data Collection, Sampling, and Recruitment

As to step 3, we designed an online form for collecting data on what GPs perceived to be factors, events, behaviors or experiences which helped or hindered their care experience or clinical practice (using the SurveyMonkey survey application), which was available from March 12 to April 17, 2020.

The data collection strategy involved gathering sociodemographic information (age, gender, workplace province and setting, and quarantine experience) and asking three open-ended questions (on the positive and negative CIs during the pandemic and the GPs' proposals to avoid the negative CIs). Although CIT usually requires researchers to collect qualitative data through interviews, for practical reasons (i.e., the need to capture data from a geographically widely distributed population and the need to collect data in a timely manner) researchers designed a questionnaire-based CIT study as suggested elsewhere (14), in line with a qualitative research approach (15). The data were collected through the online survey platform using a purposive sample of GPs, which was obtained using snowball sampling (16). The researchers invited potential participants (from among the doctors that they knew personally who they believed would be interested in the study) via phone calls in which they explained the study aims and addressed emerging questions. Following each phone call, a weblink to a brief explanation of the study, an informed consent form, and the survey itself was sent to the potential participant (via WhatsApp^©^, text message, or email). Each respondent who agreed to participate was asked to recruit other potential participants.

The participants were requested to reflect on and identify one or more specific CIs that they perceived to be positive and one or more CIs that they perceived to be negative regarding their care provision during the pandemic, and to detail any proposals that they may have regarding how to avoid the negative CIs in the future. The survey was piloted with a convenience sample of 10 participants. Thereafter, the three open-ended questions were reformulated to enhance comprehensibility and readability, as shown in Table 1.

### Data Analysis

As to step 4, namely data analysis, before approaching the dataset, the analysts were given focused training on qualitative data analysis by a qualitative methodologist (LG). Thematic analysis (17) was used, which involved defining an analytic framework (18). This framework method is suitable for multidisciplinary teams to analyze large datasets (19, 20), as recently demonstrated (21).

The analysis involved the following steps:

- All authors extensively read all the responses to the open-ended questions.
- Four authors (Al.S., PKK, Ar.S., and MD) met for subsequent discussion sessions on the provisional themes and thematic areas.
- Based on the responses of the first 20 participants, an analytic framework was developed as follows: four authors (Al.S., PKK, Ar.S., and MD) independently labeled all the responses and then met to discuss the emerging framework, with any disagreement being resolved by another researcher (LG).
- Two authors (Al.S. and Ar.S.) applied the framework to the remaining responses to the open-ended questions.
- Researchers renamed themes to highlight hindering and facilitating factors according to the research question.
- The last stage entailed grouping themes into two main thematic categories.

Finally, as to step 5, a report of the results was shared among the team and the final interpretation of data was specifically discussed in many team meetings.

The quantitative data, including sociodemographic variables, were analyzed using SAS® 9.4 (SAS Institute Inc., Cary, NC, USA).

## Results

There were 149 GPs who responded to the survey. Their demographic data are shown in Table 2. The majority (66.4%) were aged ≤35 years (26–≥66), and 38.9% were male; 26 participants declared that they were or had been quarantined. All 149 participants completed the sociodemographic form, but 50 did not answer any of the three open-ended questions (dropout rate = 33%). Among the remaining 99 respondents, all reported at least one negative CI, while three did not report any positive CIs.

**Table 2:** Sociodemographic profile of respondents by geographical area.

		**Northern regions**	**Central regions**	**Southern regions**	**Total**
Age	26–35	48 (32.21%)	20 (13.42%)	31 (20.81%)	99 (66.44%)
	36–45	5 (3.36%)	4 (2.68%)	6 (4.03%)	15 (10.07%)
	46–55	5 (3.36%)	1 (0.67%)	4 (2.68%)	10 (6.71%)
	56–65	12 (8.05%)	2 (1.34%)	6 (4.03%)	20 (13.42%)
	66+	2 (1.34%)	2 (1.34%)	1 (0.67%)	5 (3.36%)
	Total	72 (48.32%)	29 (19.46%)	48 (32.21%)	149 (100.00%)
Gender	F	44 (29.53%)	18 (12.08%)	27 (18.12%)	89 (59.73%)
	M	27 (18.12%)	11 (7.38%)	20 (13.42%)	58 (38.93%)
	Other	1 (0.67%)	0	1 (0.67%)	2 (1.34%)
	Total	72 (48.32%)	29 (19.46%)	48 (32.21%)	149 (100.00%)
Work setting	General Practice	57 (38.26%)	20 (13.42%)	20 (13.42%)	97 (65.10%)
	Out of Hours	9 (6.04%)	7 (4.70%)	20 (13.42%)	36 (24.16%)
	GP in training	5 (3.36%)	1 (0.67%)	5 (3.36%)	11 (7.38%)
	Prison	0	0	1 (0.67%)	1 (0.67%)
	Other	1 (0.67%)	1 (0.67%)	2 (1.34%)	4 (2.68%)
	Total	72 (48.32%)	29 (19.46%)	48 (31.21%)	149 (100.00%)
Quarantined	Yes	11 (7.38%)	5 (3.36%)	10 (6.71%)	26 (17.45%)
	No	61 (40.94%)	24 (16.11%)	38 (25.5%)	123 (82.55%)
	Total	72 (48.32%)	29 (19.46%)	48 (31.21%)	149 (100.00%)

**Figure 1:**
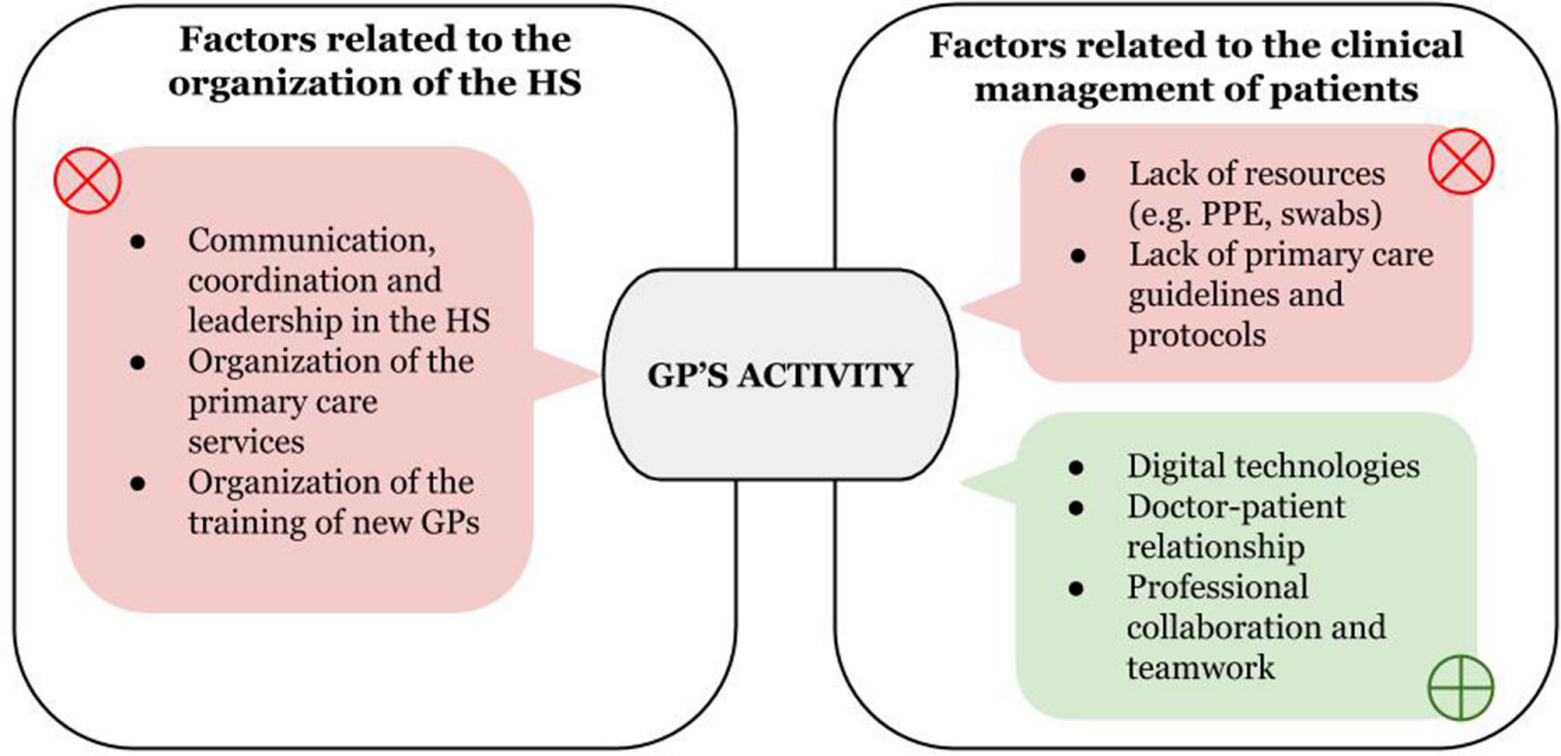
Summary of findings–themes that impacted on GPs care experiences according to study participants. Figure shows the factors emerged from the analysis that helped (green) or hindered (red) the GPs' activity. These factors were related to two main domains: the organization of the healthcare system (HS) and the clinical management of patients. GPs, General Practitioners; PPE, personal protective equipment.

Each theme signifies a factor perceived as particularly relevant for GPs and was classified according to whether it helped or hindered their care experiences (i.e., had a positive or negative impact) during the first wave of the COVID-19 pandemic. Two main thematic categories emerged: factors related to the organization of the healthcare system (HS) and factors related to the clinical management of patients, whose summary is shown in Figure 1.

### Factors Related to the Organization of the HS

Participants reported many CIs related to the overall organization of the HS: a lack of communication, coordination, and leadership of the services in charge of the crisis' management as well as a lack of organization of primary care services and training of new GPs impacted negatively. GPs perceived huge difficulties in performing public health responsibilities that could contain the epidemic.

#### Communication, Coordination, and Leadership in the HS

The GPs reported that the poor coordination and communication among and within services was one of the leading causes of the system's inflexibility and inefficiency to the detriment of patient care and effectiveness regarding containing the epidemic.

“It became clear to me the impossibility of applying our job to reality (…): fragmentation, lack of sharing and collaboration, lack of communication, abandonment, inexperience, and incapacity, the non-evidence-based practice.” (39)

“There was no coordination (…) we received directives that an hour later were denied by the other department.” (52)

The respondents indicated a lack of leadership as a trigger for the inadequacy of the services that were coordinating the response to the pandemic.

“I am in the public health service (…), I have received consciously erroneous instructions from my superiors (…). They provided handwritten, unsigned, and unrecorded instructions in blatant contradiction to ministerial provisions (…) We work only with pen and paper, papers are repeatedly photocopied and distributed in different places until they are lost, so that mistakes of the individuals can hide the intention of the organization to cover up.” (31)

“There is no clear organization of services; there is no leadership. The indications given are schizophrenic (…)” (37)

A significant system's fragmentation among regions and provinces emerged from the analysis of the respondents' experiences, which led to criticalities in patient care, disappointment, and confusion.

“I would like to point out the lack of coordination […] of two neighboring health districts, which follow different working criteria. This generated in me—and in the patient—false expectations, confusion, and disappointment.” (100)

The respondents reported that public health services struggled to take charge of and to manage suspected cases, which led to the spread of the infection and the overload of the healthcare services.

“There are no swabs. […] in the initial chaos there was no adequate surveillance. Wards, Emergency Departments, hospitals… are collapsing.” (67)

#### Organization of the Primary Care Services

Regarding the organization of the primary care service and its relationship with the rest of the National Health Service (NHS), participants reported that the importance of primary care was underestimated with a hospital-centric organization of care that contributed to the system's overload.

“The total lack of preparation to face the biggest emergency in the area since the Second World War with an excessively hospital-centered vision caused the wards to become saturated within a week.” (27)

“Much more attention is needed on primary care, which in this emergency has been abandoned to give resources to the hospital.” (73)

*“I suffer every day when I see how the current organization of Primary Care is tragically inadequate and unable to face this challenge and all the other challenges that had been posed in recent decades […]*.” *(125)*

In this context, many CIs revealed that a common experience among participants was a sense of isolation, uselessness, and lack of protection.

“No one protects us.” (13)

“I would like to be more useful in this situation, but I feel alone, like a hamster running in a wheel. I would like to act and make sense of my actions; instead, it seems to me I'm not moving an inch forward.” (107)

However, some respondents proposed that their professional roles could be developed during the pandemic, with the organizational advancement of the primary care service being fostered.

“GPs should have a stable, recognized, and integrated role in the organization of the NHS.” (3)

#### Organization of the Training of New GPs

According to the participants, the pandemic also impacted the training of new GPs. The respondents perceived that the training programs were run by “bureaucratic” structures that were unable and uninterested in training them.

“Due to the SARS-Cov2 epidemic, my training was interrupted. We were basically left to ourselves by those who should have organized it: administrative staff, teaching coordinators, and regional representatives.” (109)

“As a GP trainee, I found that course coordinators were completely unable to reschedule internships and reallocate the trainees […] [During that time] I received information and updates exclusively through unofficial channels unrelated to the GP course.” (56)

The reported lack of organization of GP courses emerged as a health risk for trainees: in some cases, trainees were told to begin internships without being provided with PPE.

“As a trainee, I was quarantined because I came into close contact with a colleague who tested positive.” (10)

“I was told to begin a hospital internship […]. I felt a constant risk related to generating gatherings; we had no PPE available.” (56)

Some participants proposed possible solutions to overcome this lack of organization and to avoid negative experiences in the future.

“The GP course should become a real course with a defined role, and it should be recognized by institutions as a specialization course like any other.” (66)

### Factors Related to the Clinical Management of Patients

Several CIs showed how the lack of resources (e.g., PPE, swabs) and of clinical guidance affected their daily practice while digital technologies, the doctor-patient relationship, and professional collaboration helped to overcome difficulties in patients care.

#### Lack of Resources

Participants reported huge difficulties in the management of their patients due to the lack of resources. Many participants reported that they were not provided with PPE. This was perceived to be an obstacle to the delivery of appropriate care to both COVID-19 and non-COVID-19 patients.

“I regret not being able to give my full contribution due to the lack of adequate PPE.” (115)

“Hearth attacks and strokes that probably have occurred and have remained unrecognized because we do not have PPE to protect ourselves…” (52)

The lack of PPE led to a sense of loneliness, vulnerability, and to psychological distress.

“During a shift in the GP out-of-hours (OoH) Service I had to visit a suspected Covid-19 patient without all the PPE (except for a surgical mask and a pair of gloves). This showed me all the vulnerability, anxiety, fear, the sense of loneliness and the lack of preparation (even psychological) for these events.” (59)

Moreover, the scarcity of PPE generated conflicts over its allocation and the responsibility for its provision.

“A colleague was infected because the head physician denied him even surgical masks.” (29)

“ I was not provided with a mask with a filter or glasses/visor despite the fact that I had to visit the patient. Instead, the paramedics […] were fully equipped with PPE.” (63)

Alongside PPE, the lack of other diagnostic equipment (such as swabs), hospital beds, therapeutics (such as oxygen tanks) and even telephone lines were reported as a criticality that negatively impacted the possibility of respondents engaging in their daily work to assure continuity of care.

“77-year-old patient in a nursing home […] he was not taken to the hospital and he died after 2 days… the patient was a suspected case […] he was not even swabbed” (14)

“I cry when I think of the call to a lady just 50 years old: I am at home, they gave me oxygen, I ran out of oxygen […] I struggle to breathe, I don't want to go to the emergency department, I'm afraid, I'm afraid to die because they have no beds.” (67)

“We spent 10 days looking for oxygen tanks throughout the province of [OMISSIS] for emergency therapy while people saturated at 82%. A real nightmare.” (52)

“In my office we have only one telephone line where patients can call (for a city of 150,000 inhabitants). It rings continuously.” (106)

#### Lack of Primary Care Guidelines and Protocols

Another factor that was mentioned in many of the CIs as critical for care delivery to patients was the lack of guidelines and protocols for primary care drawn up by authoritative and respected sources.

“I had the impression that primary care was left without a leader—without a single authoritative voice from a scientific point of view.” (20)

“I would try to strengthen the primary care service, outlining guidelines so that we know what to do.” (76)

The absence of clear guidelines and authoritative sources of knowledge, in the view of some participants, led space to differences and divergencies in the health care professionals' behaviors and views, affected teamwork, and hindered access to care.

“The different ideas about work management—even more when clear guidelines were lacking and in such a delicate phase for everyone—is making teamwork unsustainable […].” (8)

#### Digital Technologies

In this context of lack of resources and clear protocols, the respondents felt positively about the digital technologies adopted for patient consultations such as electronic prescriptions.

“I was also surprised by the rapid possibility of making electronic prescriptions accessible to patients directly from the pharmacy.” (17)

Many other positive CIs regarding telephone-based care were reported to have occurred during the process of adaptation to the pandemic situation: the possibility of remotely taking care for patients and giving emotional support to those in isolation.

“The rediscovered importance of words, of a telephone conversation that becomes an essential connection, and which is able to concentrate all the possible humanity, closeness and help.” (39)

“Every day I called them, I entered their homes, I saw their eyes, I evaluated their breathing. […] I have been living with them for these 20 days.” (42)

#### Doctor-Patient Relationship

Participants reported that relational aspects of their work (relationships with patients and colleagues or other healthcare professionals as well as the attitudes and behaviors of patients) had a role in their clinical practice.

Mainly, what facilitated them in facing their work in the context of the COVID-19 emergency was the doctor/patient relationship. They described events in which patients expressed gratitude, understanding and appreciation to their efforts.

“The relief of this person in being reassured after having been visited… She thanked me. This is to date one of the very few positive experiences of this period.” (8)

“Another thing that surprised me positively was hearing a patient asking me: ‘Before starting, doctor, first of all tell me how you are, because right now you are the people who most need to hear this asked. And maybe nobody does.”’ (25)

According to some CIs, GPs' clinical practice was also supported by the behavior of their patients who accepted the access limitation to health services during the first phase of the emergency. The GPs reported a relatively high level of compliance to the rules.

“The only positive aspect that I can find right now is that patients […] have understood and have used the service in an appropriate way.” (10)

“[A positive experience has been] The understanding of my elderly homebound patients when I had to cancel the planned home visits and the collaboration of their families in helping them and providing medicines for them.” (29)

#### Professional Collaboration and Teamwork

Also, relationships with GP colleagues, with doctors working in different outpatient settings, with those in hospitals, and with other healthcare professionals had a facilitating role for our participants' daily work.

In this context, professional collaboration served as emotional support and allowed GPs to overcome the sense of loneliness and the emotional burden caused by the epidemic.

“I observe mutual help (an attitude that was not always previously present). Despite the forced distance, we did not feel alone, or at least, it is true for me. […]” (18)

“Another consideration concerns colleagues: I have discovered (or perhaps was confirmed) that some of them may be your strength, your constant mirror, your vent valve, the eyes that most offer you an understanding in such an emotionally and professionally heavy period.” (8)

”*The need to talk to colleagues more often, every day, even several times a day to share what is happening […] the feeling of being alone amplifies distances. Colleagues, at this moment, save you more than anyone else.” (114)*

The teamwork with doctors and other healthcare professionals was experienced as a factor that helped to overcome practical difficulties in daily work such as the lack of PPE, of official guidance and of diagnostics, and allowed for better patient management.

“We started communicating on group chats to support us and exchange information, to get masks, gowns, and oximeters. We adopted a common emergency management line.” (144)

“I immediately got in touch with my colleague in the nearby ED and my radiologist colleague and I agreed on the most appropriate and fastest path for my patient.” (25)

“If I only could have had the help of the nurse to go with PPEs to see the elderly homebound COVID-19 patient.” (22)

“[a positive experience was] the post-discharge management of a patient I have been following since March 19. I collaborated with the cardiologist and with the ADI [integrated homecare service]” (53)

## Discussion

### Summary of the Main Findings

As far as we know, this study is the first to systematically analyze GPs' experiences on the COVID-19 pandemic by employing the CIT. The surveyed GPs reported both positive and negative CIs occurring during the first peak of the outbreak.

The analysis identified factors that impacted their care experiences. The summary of findings (Figure 1) shows how these factors are related to two main domains: “organization of the HS” and “management of patients.” With regards to the first domain, it is notable that we could not identify any factor in the data that could have favorably impacted the GPs' work. Conversely, the GPs in our study experienced a lack of organization within the NHS, a lack of interinstitutional cooperation, a lack of leadership, and a lack of clear communication at all levels of the emergency response system. They described a sense of abandonment and solitude and felt that they were not part of a system that could sustain them during the emergency. The lack of reliable clinical and organizational guidance was a major challenge as was the absence of commitment regarding the teaching and supervision of GP trainees. Concerning the second domain (“clinical management of patients”), digital technologies, and meaningful and empathic doctor/patient relationships–along with collaborations with doctors (specialists, GPs) and other healthcare professionals–were described as factors that helped the GPs to cope with the organizational and emotional challenges of being on the frontline. On the contrary, lack of resources (e.g., PPE, swabs) and of reliable guidance affected patient management.

### Comparison to Existing Literature

Study participants reported a significant lack of structured coordination that resulted in communication problems between the different health services and in bureaucratic obstacles. The published literature shows that similar issues have been experienced in other healthcare systems. In the US, the fragmentation of primary care and its weak connection to the emergency response infrastructure has been an obstacle to an efficient response to the pandemic (22). In the UK, even though primary care is a cornerstone of the NHS, the links between primary care and Public Health England's broader preventive activities have been reported as unclear (7). This disconnection was also highlighted by several participants in our study and seems to be widespread. It is even more surprising in Italy because there is a well-established publicly funded surveillance network for influenza and influenza-like illnesses that involves GPs and primary care pediatricians since 1999 (the Italian Influenza Surveillance Network, InfluNet) (23). Despite the recognition that the structured involvement of GPs in infectious disease surveillance and control measures is an essential element of pandemic preparedness (24), this network only began to be involved in COVID-19 surveillance on October 14, 2020 (25).

Many GPs in our study reported that the role of primary care was underestimated—much more importance was given to hospital care—and they felt unprotected and isolated. In fact, most Italian GPs still work single-handedly in solo practices that are somewhat isolated from the rest of the NHS (26). Indeed, a recent study showed how Italian GPs represent 44.1% of the total COVID-19-related deaths among doctors, and organizational issues (i.e., working alone), along with the lack of PPE, were proposed as explanations for the high burden suffered by GPs (27). Moreover, in many regions of Italy such as Lombardy, regional policies fostered a strong hospital-centric organization while primary care has been underfinanced for many years (28). The autonomy of the Italian regions in organizing their healthcare systems led to different healthcare models across the country. To date, no published study has yet addressed the impact of these different care organizations (i.e., hospital vs. primary care centered) in Italy.

Regarding training of new GPs, courses were perceived as poorly organized and led in a bureaucratic fashion. Respondents reported that this negatively impacted their training and their safety during the first peak of the outbreak. These findings are consistent with previously published studies, which showed how GP trainees in Italy are enrolled in non-academic regional courses of questionable quality (29, 30). In fact, during the first months of the outbreak, many GP traineeship activities were stopped due to the lack of PPE, which was not available for trainees undertaking purely observational internships. Since then, trainees have been employed in GP out-of-hours services in the COVID-19 special units and to replace regular GPs; a government decree stated that the hours worked in these services would be recognized as part of the traineeships despite the fact that they occurred without clinical supervision (31). GP trainees therefore became part of the paid GP workforce. Further research on the quality of GP training in Italy is needed to overcome these critical issues (29, 30). This knowledge will explain how training has been impacted by COVID-19.

Difficulties in obtaining reliable information, guidelines, and protocols on patient management were described. This finding is consistent with influenza pandemic research, which highlighted that there were multiple information sources with conflicting recommendations and a lack of guidelines tailored to primary care providers (32). As stated elsewhere, primary care practice guidelines need to be underpinned by evidence collected in primary care settings (33, 34). There are no primary care departments in Italian Universities, and it is not possible for Italian GPs to pursue a PhD in general practice. Moreover, local or national networks with accessible research databases are missing; the only Italian general practice research database is owned by a private company, and data are not accessible to independent epidemiological research (35). As a result, to date, the context-specific evidence needed to underpin guidelines relevant for Italian GPs is missing.

In addition, the GPs in our study reported events related to a lack of resources (such as PPE or swabs) allocated in the primary care setting. Scarcity and unequal distribution of PPE were reported previously in other countries (36–38) as well as in Italy (39). The lack of PPE was psychologically stressful for our study participants—a finding that is consistent with recent studies showing how the lack of PPE and proper safety procedures are associated with higher levels of anxiety and depression (40) while access to adequate PPE is associated with reduced psychological morbidity (41). Besides, other resources such as oxygen and swabs for COVID-19 testing were also reported as insufficiently available leading to difficulties in taking charge of patient needs. In this context, the issue of how primary care should be organized, financed, and staffed is considered one of the top ten international research priorities (42). Such studies are currently lacking in Italy and could inform policies on the most efficient allocation of resources within the NHS.

The rapid switch to remote assessment via telephone or video consultations has been perceived as generally positive. A study in the UK found that the rate of initial general practice consultations in the form of digital consultations dramatically increased between February and May 2020; the UK primary care service concurrently faced profound organizational challenges (43). No published studies have yet analyzed nor quantified these changes in Italy, and research is needed to evaluate the impact of COVID-19 on GP consultations, digital technology use, and remote assessment and on the related health outcomes of these procedures.

The GPs reported that they experienced an improvement in their relationships with their patients in terms of compliance, patient understanding, and solidarity. Similar findings in China have been reported despite the decline in doctor/patient relationships since the late 1970s (44). A possible explanation for this phenomenon is that an outbreak of the scale of COVID-19 could reduce the emotional distance between doctors and patients. In fact, the so-called hidden curriculum traditionally encourages detachment between emotions and clinical reasoning (45, 46) to preserve the objectivity of clinical judgement, i.e., avoiding the interference of the doctor's empathic concern that could affect clinical decision making (47). Doctors could have perceived increased solidarity from their patients as the doctors themselves felt closer to them because they were sharing the common concern of COVID-19. More research is needed to understand the impact of COVID-19 on empathy in medicine and, more broadly, on the doctor/patient relationship.

From the perspective of the GPs in our study, collaboration among professionals and teamwork seemed to be a valuable resource to cope with the clinical and emotional challenges that they have faced. In line with our findings, in the case of influenza outbreaks, teamwork and interprofessional collaboration were described as factors that can lead to a more adequate response (48). Healthy teams showed to be effective in preventing burnout among GPs (49), in improving professional motivation (50, 51), and patient and family-centered care (52). That said, interprofessional collaboration and teamwork is not well-established among Italian GPs (53), and further research is needed to address the impact of the working environment on mental health, safety, and care delivery of Italian GPs during the COVID-19 pandemic.

### Strengths and Limitations

Our findings need to be interpreted in light of their explorative nature. Nevertheless, they do offer a direction for policymakers and for further studies. One limitation of this study is that the data collection was via an online survey. In the CIT methodology, even if survey/online data collection is allowed, interviews are recommended to collect in-depth information (14). The risk of survey-related poor data collection was balanced by capturing a wide range of experiences and perspectives in an unexplored area of research and in a large, diverse and geographically widely distributed population. Moreover, piloting helped to identify possible problems with the interpretation of the questionnaire and with the open-ended questions being modified based on the preliminary feedbacks. Moreover, due to the open-ended nature of the questions and the fact that no personal data were collected, motivated participants were free to disclose their experiences without fear of being judged and without manipulation introduced by an external interviewer (15, 54).

The second limitation relates to the findings' generalizability. Indeed, this study followed a qualitative approach in sampling and analysis (8, 55). Bearing in mind this approach and the explorative, rather than definitive, nature of the results, the generalizability to the entire population of Italian GPs was beyond the aims of this study (56). Nonetheless, the richness of the data allowed us to acquire a meaningful picture of GPs managing the outbreak through an analysis from which the themes in common across the participants could emerge (57). The majority of the participants were from northern Italy where the pandemic started and was more threatening. This could explain the wider participation of GPs located in these regions and probably contributed to their informative responses. In addition, most of the participants were aged ≤35 years, and it is likely that our findings match the perspectives of younger GPs. Some of the participants stated that they were enrolled in a GP specialization course. This should not be interpreted as meaning that their responses do not reflect the perceptions of actual GPs because GP specialization schools were suspended shortly after the beginning of the first outbreak, and GP trainees were asked to take part in the response to the healthcare emergency becoming part of the paid GP workforce.

Regarding the analysis, the principal investigators of this study were notably GPs. As such, they may have analyzed the CIs from an emic perspective, and interpretation could have been limited. Nonetheless, every step of the analysis was performed by at least two researchers, and analytical decisions were made by reaching an agreement among an interdisciplinary team during each step. Additionally, public health experts (MFM and MGC) and GPs collaborated with two authors with no health-related background (i.e., LG, a qualitative methodologist, and Ar.S., who works as a lawyer in training with a special interest in medico-legal issues).

### Implications for Policy

This study suggests that GPs in Italy are not part of a coherent strategy that prepares the Italian primary care service for epidemic outbreaks.

Several recommendations may be drawn. Communication and coordination between primary care and public health authorities are essential and should be substantially improved. Funding should be allocated for the integration of primary care and public health services, and structured teamwork should be enhanced through shared protocols and guidelines to contain the outbreak. Efforts should be made to adequately train GPs based on national guidelines on the management of COVID-19 and non-COVID-19 patients in their setting. To inform these guidelines in the longer term, primary care research is a necessity for the Italian NHS that, in the light of this pandemic, can no longer be postponed. Unfortunately, there is currently no publicly funded and institutional general practice research in Italy but it is urgently needed to produce context-specific evidence (33, 58) to help GPs in their daily practice and to effectively train the next generation of primary care doctors.

### Implications for Further Research

Research is needed to ascertain how the Italian primary care service and, more broadly, European primary care services are coping with the pandemic. With Europe facing a second wave of COVID-19, a follow-up study could be useful to ascertain whether and how GPs' experiences change over time. To further enhance the credibility of our findings, themes that emerged in this study, such as the impact of COVID-19 on the doctor–patient relationship, should be more extensively explored, e.g., through semi-structured and in-depth interviews with doctors and patients. Moreover, quantitative studies should be performed to ascertain the generalizability of the results of the present study. Moreover, participatory methodologies, such as participatory action research, could be applied to develop an understanding of the collective experience of this pandemic and to enable healthcare professionals to cope effectively with the challenges they face during health emergencies.

## Data Availability Statement

The raw data supporting the conclusions of this article will be made available by the corresponding author under reasonable request.

## Ethics Statement

No detailed personal data or any identifying information (e.g., names) were collected during the study. Before data collection, the ethics committee of the Local Health Authority of Area Vasta Emilia Nord (AVEN) was approached, and the authors were advised that, according to Italian law, formal ethical approval was not necessary.

## Author Contributions

PKK, AlS, and LG conceptualized the study in several discussions that involved all the authors. MFM and MGC were consulted as experts in public health research issues. PKK, AlS, MD, and AM, with the collaboration of the Research Team of the Giotto Movement, performed data collection. PKK, AlS, ArS, and MD performed the qualitative data analysis under the supervision of LG while iteratively discussing the emerging themes with MFM, AM, and MGC. MFM and MGC performed the quantitative analyses of the dataset. PKK and LG drafted the manuscript, which was discussed among all the authors, who all made relevant amendments and approved the final version of the manuscript.

## Conflict of Interest

The authors declare that the research was conducted in the absence of any commercial or financial relationships that could be construed as a potential conflict of interest.

## References

[1] Ministero della Salute Covid-19 - Situazione in Italia. (2020). Available online at: http://www.salute.gov.it/portale/nuovocoronavirus/dettaglioContenutiNuovoCoronavirus.jsp?area=nuovoCoronavirus&id=5351&lingua=italiano&menu=vuoto (accessed December 27, 2020).

[2] Gazzetta Ufficiale della Repubblica Italiana Decreto del Presidente del Consiglio dei Ministri 9 Marzo 2020, Gazzetta Ufficiale Serie Generale n. 62 del 09/03/2020. (2020). Available online at: https://www.gazzettaufficiale.it/eli/id/2020/03/09/20A01558/sg (accessed October 29, 2020).

[3] Gazzetta Ufficiale della Repubblica Italiana Decreto del Presidente del Consiglio dei Ministri 11 Marzo 2020, Gazzetta Ufficiale Serie Generale n. 64 del 11/03/2020. (2020). Available online at: https://www.gazzettaufficiale.it/eli/id/2020/03/09/20A01558/sg (accessed October 29, 2020).

[4] Liu Q Luo D Haase JE Guo Q Wang XQ Liu S. The experiences of health-care providers during the COVID-19 crisis in China: a qualitative study. Lancet Global Health. (2020) 8:e790–8. 10.1016/S2214-109X(20)30204-7 32573443 PMC7190296

[5] World Health Organization Primary Health Care and Health Emergencies. Geneva: World Health Organization (2018). p. 13. Available online at: https://www.who.int/docs/default-source/primary-health-care-conference/emergencies.pdf?sfvrsn=687d4d8d_2 (accessed October 15, 2020).

[6] Sarti TD Lazarini WS Fontenelle LF Almeida A. What is the role of Primary Health Care in the COVID-19 pandemic? Epidemiol Serv Saude. (2020) 29:e2020166. 10.5123/s1679-49742020000200024 32348404

[7] Park S Elliott J Berlin A Hamer-Hunt J Haines A. Strengthening the UK primary care response to covid-19. BMJ. (2020) 370:m3691. 10.1136/bmj.m3691 32978177

[8] Flanagan JC The critical incident technique. Psychol Bull. (1954) 51:327–58. 10.1037/h0061470 13177800

[9] Schluter J Seaton P Chaboyer W. Critical incident technique: a user's guide for nurse researchers. J Adv Nurs. (2008) 61:107–14. 10.1111/j.1365-2648.2007.04490.x 18173737

[10] Viergever RF The critical incident technique: method or methodology? Qual Health Res. (2019) 29:1065–79. 10.1177/1049732318813112 30600767

[11] Rodríguez-Rey R Palacios A Alonso-Tapia J Pérez E Álvarez E Coca A. Posttraumatic growth in pediatric intensive care personnel: dependence on resilience and coping strategies. Psychol Trauma. (2017) 9:407–15. 10.1037/tra0000211 27929306

[12] Butterfield LD Borgen WA Amundson NE Maglio A-ST Fifty years of the critical incident technique: 1954-2004 and beyond. Qual Res. (2005) 5:475–97. 10.1177/1468794105056924

[13] Danielis M Chiaruttini S Palese A Unplanned extubations in an intensive care unit: findings from a critical incident technique. Intensive Crit Care Nurs. (2018) 47:69–77. 10.1016/j.iccn.2018.04.012 29776707

[14] Serrat O The critical incident technique. In: Serrat O, editor. Knowledge Solutions: Tools, Methods, and Approaches to Drive Organizational Performance. Singapore: Springer Singapore (2017). p. 1077–83. 10.1007/978-981-10-0983-9_123

[15] Braun V Clarke V Boulton E Davey L McEvoy C. The online survey as a qualitative research tool. Int J Soc Res Methodol. (2020) 1–14. 10.1080/13645579.2020.180555027535555

[16] Noy C Sampling knowledge: the hermeneutics of snowball sampling in qualitative research. Int J Soc Res Methodol. (2008) 11:327–44. 10.1080/13645570701401305

[17] Braun V Clarke V Using thematic analysis in psychology. Qual Res Psychol. (2006) 3:77–101. 10.1191/1478088706qp063oa

[18] Ritchie CS Leff B Garrigues SK Perissinotto C Sheehan OC Harrison KL. A quality of care framework for home-based medical care. J Am Med Dir Assoc. (2018) 19:818–23. 10.1016/j.jamda.2018.05.020 30056010 PMC6392035

[19] Gale NK Heath G Cameron E Rashid S Redwood S. Using the framework method for the analysis of qualitative data in multi-disciplinary health research. BMC Med Res Methodol. (2013) 13:117. 10.1186/1471-2288-13-117 24047204 PMC3848812

[20] Pope C Ziebland S Mays N. Analysing qualitative data. BMJ. (2000) 320:114–6. 10.1136/bmj.320.7227.114 10625273 PMC1117368

[21] Verhoeven V Tsakitzidis G Philips H Van Royen P. Impact of the COVID-19 pandemic on the core functions of primary care: will the cure be worse than the disease? A qualitative interview study in Flemish GPs. BMJ Open. (2020) 10:e039674. 10.1136/bmjopen-2020-039674 32554730 PMC7306272

[22] Roehr B. Covid-19 is threatening the survival of US primary care. BMJ. (2020) 369:m2333. 10.1136/bmj.m2333 32571895

[23] Gabutti G Guido M Quattrocchi M Zizza A De Donno A Gasparini R. Surveillance of influenza in Apulia, Italy, 1999-2000, 2000-2001, 2001-2002, and 2002-2003 seasons. Med Mal Infect. (2004) 34:469–76. 10.1016/S0399-077X(04)00145-3 15747472 PMC7126099

[24] Patel MS Phillips CB Pearce C Kljakovic M Dugdale P Glasgow N. General practice and pandemic influenza: a framework for planning and comparison of plans in five countries. PLoS ONE. (2008) 3:e2269. 10.1371/journal.pone.0002269 18509538 PMC2386973

[25] Ministero della Salute Stagione influenzale 2020-2021, protocollo operativo InfluNet & CovidNet. (2020). Available online at: http://www.salute.gov.it/portale/nuovocoronavirus/dettaglioNotizieNuovoCoronavirus.jsp?lingua=italiano&menu=notizie&p=dalministero&id=5122 (accessed October 29, 2020).

[26] Garattini L Padula A. English and Italian national health services: time for more patient-centered primary care? Eur J Intern Med. (2018) 57:19–21. 10.1016/j.ejim.2018.09.013 30279035

[27] Modenese A Gobba F. Increased risk of COVID-19-related deaths among general practitioners in Italy. Healthcare (Basel). (2020) 8:155. 10.3390/healthcare8020155 32503304 PMC7349697

[28] Golinelli D Bucci A Adja KYC Toscano F. Comment on: “The Italian NHS: What Lessons to Draw from COVID-19?”. Appl Health Econ Health Policy. (2020) 18:739–41. 10.1007/s40258-020-00608-2 32833141 PMC7443390

[29] Cegolon L Heymann W. International primary care snapshot: academic primary care in Italy. Br J Gen Pract. (2016) 66:34. 10.3399/bjgp16X683197 26719467 PMC4684012

[30] Cegolon L Heymann WC Lange JH Xodo C. Improving Italian general practice training: the role of academia. BJGP Open. (2017) 1:bjgpopen17X100989. 10.3399/bjgpopen17X100989 30564670 PMC6169961

[31] Gazzetta Ufficiale della Repubblica Italiana D.L. 9/03/2020 n. 14: disposizioni urgenti per il potenziamento del Servizio sanitario nazionale in relazione all'emergenza COVID-19, Articolo 14. (2020). Available online at: https://www.gazzettaufficiale.it/eli/id/2020/03/09/20G00030/sg (accessed October 29, 2020).

[32] Kunin M Engelhard D Piterman L Thomas S. Response of general practitioners to infectious disease public health crises: an integrative systematic review of the literature. Disaster Med Public Health Prep. (2013) 7:522–33. 10.1017/dmp.2013.82 24274132

[33] De Maeseneer JM van Driel ML Green LA van Weel C. The need for research in primary care. Lancet. (2003) 362:1314–9. 10.1016/S0140-6736(03)14576-X 14575979

[34] De Maeseneer JM De Sutter A. Why research in family medicine? A superfluous question. Ann Fam Med. (2004) 2(Suppl. 2):S17–22. 10.1370/afm.148 15655082 PMC1466767

[35] Fabiani L Scatigna M Panopoulou K Sabatini A Sessa E Donato F. Health Search–Research Institute of the Italian Society of General Practice: the creation of a research database in general practice. Epidemiol Prev. (2004) 28:156–62. 15532872

[36] Dyer C. Covid-19: doctors make bid for public inquiry into lack of PPE for frontline workers. BMJ. (2020) 369:m1905. 10.1136/bmj.m1905 32398219

[37] Iacobucci G. Covid-19: Doctors still at “considerable risk” from lack of PPE, BMA warns. BMJ. (2020) 368:m1316. 10.1136/bmj.m1316 32234713

[38] Ranney ML Griffeth V Jha AK. Critical supply shortages — the need for ventilators and personal protective equipment during the Covid-19 pandemic. N Engl J Med. (2020) 382:e41. 10.1056/NEJMp2006141 32212516

[39] Savoia E Argentini G Gori D Neri E Piltch-Loeb R Fantini MP. Factors associated with access and use of PPE during COVID-19: a cross-sectional study of Italian physicians. PLoS ONE. (2020) 15:e0239024. 10.1371/journal.pone.0239024 33044978 PMC7549784

[40] Smith PM Oudyk J Potter G Mustard C The Association between the Perceived Adequacy of Workplace Infection Control Procedures and Personal Protective Equipment with Mental Health Symptoms: A Cross-sectional Survey of Canadian Health-care Workers during the COVID-19 Pandemic: L'association entre le caractère adéquat perçu des procédures de contrôle des infections au travail et de l'équipement de protection personnel pour les symptômes de santé mentale. Un sondage transversal des travailleurs de la santé canadiens durant la pandémie COVID-19. Can J Psychiatry. (2020). 10.1177/0706743720961729 PMC7509238 32957803

[41] Kisely S Warren N McMahon L Dalais C Henry I Siskind D. Occurrence, prevention, and management of the psychological effects of emerging virus outbreaks on healthcare workers: rapid review and meta-analysis. BMJ. (2020) 369:m1642. 10.1136/bmj.m1642 32371466 PMC7199468

[42] O'Neill B Aversa V Rouleau K Lazare K Sullivan F Persaud N. Identifying top 10 primary care research priorities from international stakeholders using a modified Delphi method. PLoS ONE. (2018) 13:e0206096. 10.1371/journal.pone.0206096 30359391 PMC6201922

[43] Joy M McGagh D Jones N Liyanage H Sherlock J Parimalanathan V. Reorganisation of primary care for older adults during COVID-19: a cross-sectional database study in the UK. Br J Gen Pract. (2020) 70:e540–7. 10.3399/bjgp20X710933 32661009 PMC7363277

[44] Gao B Dong J. Does the impact of COVID-19 improve the doctor-patient relationship in China? Am J Med Sci. (2020) 360:305–6. 10.1016/j.amjms.2020.05.039 32563570 PMC7831870

[45] Shapiro J. Perspective: does medical education promote professional alexithymia? A call for attending to the emotions of patients and self in medical training. Acad Med. (2011) 86:326–32. 10.1097/ACM.0b013e3182088833 21248595

[46] Mahood SC. Medical education: beware the hidden curriculum. Can Fam Physician. (2011) 57:983–5. 21918135 PMC3173411

[47] Silva JV Carvalho I. Physicians experiencing intense emotions while seeing their patients: what happens? Perm J. (2016) 20:15–229. 10.7812/TPP/15-229 27479947 PMC4991912

[48] Shortridge KF Peiris JSM Guan Y. The next influenza pandemic: lessons from Hong Kong. J Appl Microbiol. (2003) 94:70–9. 10.1046/j.1365-2672.94.s1.8.x 12675938

[49] Galleta-Williams H Esmail A Grigoroglou C Zghebi SS Zhou AY Hodkinson A. The importance of teamwork climate for preventing burnout in UK general practices. Eur J Public Health. (2020) 30:iv36–8. 10.1093/eurpub/ckaa128 32894291 PMC7526765

[50] Matziou V Vlahioti E Perdikaris P Matziou T Megapanou E Petsios K. Physician and nursing perceptions concerning interprofessional communication and collaboration. J Interprof Care. (2014) 28:526–33. 10.3109/13561820.2014.934338 25003547

[51] Josi R Bianchi M Brandt SK. Advanced practice nurses in primary care in Switzerland: an analysis of interprofessional collaboration. BMC Nurs. (2020) 19:1. 10.1186/s12912-019-0393-4 31908597 PMC6941298

[52] Fox S Gaboury I Chiocchio F Vachon B. Communication and interprofessional collaboration in primary care: from ideal to reality in practice. Health Commun. (2021) 36:125–135. 10.1080/10410236.2019.1666499 31580162

[53] Bonciani M Barsanti S Murante AM. Is the co-location of GPs in primary care centres associated with a higher patient satisfaction? Evidence from a population survey in Italy. BMC Health Serv Res. (2017) 17:248. 10.1186/s12913-017-2187-2 28376886 PMC5379750

[54] Chenail RJ Interviewing the investigator: Strategies for addressing instrumentation and researcher bias concerns in qualitative research. Qual Rep. (2011) 16:255–62.

[55] Jones MP. Not how many but why. A qualitative approach to customer relations. Health Serv Manage. (1988) 84:175–7. 10291465

[56] Cutcliffe JR McKenna HP. Establishing the credibility of qualitative research findings: the plot thickens. J Adv Nurs. (1999) 30:374–80. 10.1046/j.1365-2648.1999.01090.x 10457239

[57] Sandelowski M. Sample size in qualitative research. Res Nurs Health. (1995) 18:179–83. 10.1002/nur.4770180211 7899572

[58] Kurotschka PK Serafini A Massari M Da Cas R Figueiras A Forte V. Broad Spectrum project: factors determining the quality of antibiotic use in primary care: an observational study protocol from Italy. BMJ Open. (2020) 10:e038843. 10.1136/bmjopen-2020-038843 32636291 PMC7342852

