# Supplementary material for: General Practitioners' Experiences During the First Phase of the COVID-19 Pandemic in Italy: A Critical Incident Technique Study
Source: Front Public Health. 2021 Feb 3;9:623904. doi: 10.3389/fpubh.2021.623904 (PMC7888233; doi:10.3389/fpubh.2021.623904)
Supplement: Supplementary file 1 [file Data_Sheet_1.docx]

**Supplementary file 1, Table S1: Quotations related to the domain Organization of the Healthcare System**

Â

| **Themes** | **Quotations** |
| --- | --- |
| **Communication, coordination and leadership in the HS** | *“To my request to send official communications, I was answered that ‘there is a lot to do and no time to send official communications to doctors.’” (9)*  *"I am in the public health service, as part of the GP specialty training. I answer calls from private citizens and if appropriate I request a swab and I issue a mandatory home isolation order. I have had no training and I have received consciously erroneous instructions from my superiors NOT to request swabs, so that their inability to manage the situation was hidden behind the [individual] mistakes of the prescriber. They provided handwritten, unsigned, and unrecorded instructions in blatant contradiction to ministerial provisions. To each question, no answer. One day, IÂ was told to speak with colleagues from [OMISSIS] to draft a protocol, but in the meantime other superiors have drawn up one, which they sent to the Crisis Unit for approval. The Crisis Unit never approved it for obvious mistakes, but they put it in circulation after a few days, unsigned and unofficially registered. In this protocol, the swab is not even mentioned once. [. . .] Even those involved in surveillance have not received instructions and are not healthcare professionals, so that they do not ask questions and take measures when the "surveilled patient" (close contact of a suspicious or ascertained case) get sick. We work only with pen and paper, papers are repeatedly photocopied and distributed in different places until they are lost, so that mistakes of the individuals can hide the intention of the organization to cover up and not to control the situations. There is no official channel for communication with GPs, which are not informed or provided with instructions about the procedures. While I am there, I feel guiltily useless. I have convinced my colleagues at least to ask for swabs, but they cannot carry them out from an organizational perspective. Every day I check for new document, because we are never told anything. Moreover, the staff walks around without masks and without gloves and the staff coordinators are perpetually meeting all together.” (31)*  *“There is no clear organization of services; there is no leadership. The indications given are schizophrenic; every single professional unit does its own thing and there is no clarity of paths. This should make us reflect and lead, as soon as possible, to a profound reorganization of territorial assistance, so unsustainable.” (37)*  *“I felt helpless, alone, facing the unknown. I felt the agitation of my mind and that of some colleagues close to me, but after an initial moment of euphoria in which I felt I could finally give space to concrete actions and good practices. It became clear to me the impossibility of applying our job to reality, the lack of communication between the multiple actors of the NHS [National Health Service]. I felt the weight of all that I cannot bear about our healthcare: fragmentation, lack of sharing and collaboration, lack of communication, abandonment, inexperience, and incapacity, the non-evidence-based practice.” (39)*  *"The Local Health Service did not have an emergency plan; I never saw the risk manager at any meeting. They failed to understand the needs of physicians, there was no top-down coordination between primary care and infectious diseases, so we received directives that an hour later was denied by the other department. How can we work like this? I have seen managers get lost behind unnecessary issues and lose sight of the main objectives. Nor have they been able to define the essential priorities and needs of physicians and of the population. This is because they are strongly distant from the territory itself and uninterested to a dialog." (52)*  *“If you are responsible for organizing a service, you must ask for help. Faced with an emergency, an epidemic that you have never faced, do not be obtuse. [. . .] The reaction should be collective. There should be a dialog among services. There must be a common line. There can be no guidelines stolen from the bordering region and somewhat blurred after weeks from the beginning. We have been too slow. The machine is not efficient.” (67)*  *"[. . .] That day he had been contacted by the public health service, but either he did not understand, or the line had fallen, he was still in the house, in isolation, with his wife's corpse. I phoned the public health service to try unblocking the situation, but because of communication problems between the different emergency management offices and medico-legal problems regarding who was required to know the swab’s result [. . .], I could not help" (70)*  *[. . .] I had agreed [with the Public Health service of [OMISSIS] for a patient resident in the province of [OMISSIS] to be swabbed due to highly suspicious symptoms of COVID-19. Subsequently, this agreement was canceled, as it was not considered to belong to the territory of [OMISSIS]. I had to resume contact with the Public Health Service of [OMISSIS], which, however, denied the swab due to lack of epidemiological criteria (no certain contact with COVID-19 + patients). Therefore, I want to highlight the lack of coordination [â€¦] of two neighboring health districts, which follow different working criteria. This generated in me and in the patient false expectations, confusion, and disappointment.” (100)* |
| **Organization of primary care services** | *“GPs should have a stable, recognized and integrated role in the organization of the NHS.” (3)*  *“No one protects us” (13)*  *"The total lack of preparation to face the biggest emergency in the area since the Second World War, with an excessively hospital-centered vision, which led the wards to become saturated within a week." (27)*    *"As GPs we were alone, without PPE supplies, with the obligation to manage the unmanageable. [There was] no help. [. . .] The only positive note was the mutual help between GPs.” (49)*  *"Much more attention is needed on primary care, which in this emergency has been abandoned to give resources to the hospital." (73)*  *“I would like to be more useful in this situation, but I feel alone, like a hamster running in a wheel. I want to act and make sense of my actions; instead, it seems to me I am not moving an inch forward.” (107)*  *“I replaced a GP from March 9 to March 13; in that week I had to give new rules to patients: access only by appointment, telephone triage for symptomatic patients, recipes preferably electronically and postponement of everything that was not urgent, but that was deferrable (and therefore also of all prevention). As patients adapted to these rules during the week, I felt progressively emptied of my role and progressively increasingly useless.” (118)*    *“I suffer every day when I see how the current organization of Primary Care is tragically inadequate and unable to face this challenge and all the other challenges posed in recent decades. [. . .] Unfortunately, the nurse who works with us has a 6-hour-a-week contract so I can't do much.” (125)* |
| **Organization of the training of new GPs** | *“In a word, the bureaucrats. As a general practitioner [in training] I was told that there were problems of incompatibility between the 800 euro per month I was paid to attend the [GP specialty] course and the desire I had to launch myself in the territory to assist people in emergency.” (27)*  *“As a GP trainee, I found that course coordinators were completely unable to reschedule internships and reallocate the trainees [. . .] [During that time] I received information and updates exclusively through unofficial channels unrelated to the GP course.* *I was told to begin a hospital internship (â€¦). I felt a constant risk related to generating gatherings; we had no PPE available.” (56)*  *“For three weeks they suspended all of our activities and were unable to relocate us as future general practitioners in any role, despite the emergency and the great demand for doctors. They rejected all our proposals for activities, always for bureaucratic reasons, not recognizing our role as physicians.” (66)*  *"Due to the SARS-Cov2 epidemic, my training was interrupted. We were left to ourselves by those who should have organized it, administrative staff, teaching coordinators and regional representatives." (109)* |

Â

**Supplementary file 1, Table S2: Quotations related to the domain “Clinical management of patients”**

| **Themes** | **Quotations** |
| --- | --- |
| **Lack of resources** | *"77-year-old patient in a nursing home [. . .] he was not taken to the hospital and he died after 2 days . . . The patient was a suspected case [. . .] he was not even swabbed" (14)*  *“A colleague was infected because the head physician denied him even surgical masks.” (29)*  *"We did not have access to liquid oxygen and we spent 10 days looking for oxygen tanks throughout the province of [OMISSIS] for emergency therapy, sending relatives to pharmacies to retrieve them for emergency therapy, while people saturated at 82%. A real nightmare [. . .] I also remember the lady who called desperately from [OMISSIS] because she was unable to reach the 112 [emergency number] for the long phone waits, and the mother who had repeated epileptic seizures without resuming consciousness. . . and not being able to help her because we are 70 km away and the out-of-hour (OoH) service in that area was not answering. . . or the hearth attacks and strokes that have probably occurred and have remained unrecognized because we do not have PPE to protect ourselves to go to visit people safely. . ." (52)*  *“Many cases are unmanageable with the few resources available at home, although they [patients] would not require to be hospitalized but adequate outpatient care that GPs often cannot provide due to risks posed to their own/other patients’ safety.” (55)*  *"During a shift in the GP OoH Service I had to visit a suspected Covid-19 patient, without all PPE (except for a surgical mask and a pair of gloves). This showed me all the vulnerability, anxiety, fear, the sense of loneliness and the lack of preparation (even psychological) for these events." (59)*  *"[. . .] I was not provided with a mask with a filter or glasses/visor even though I had to visit the patient. Instead, the paramedics [. . .] were fully equipped with PPE." (63)*  *“There are no swabs. The patients disperse because they are not assisted (it is absurd that the Public Health system works only if you have a piece of paper reporting the swab result in your hand). Result? The real cases are at least 10 times as many as the cases swabbed [. . .] in the initial chaos there was no adequate surveillance. The cases have multiplied. Wards, emergency departments, hospitals [. . .] are collapsing. Seriously ill patients are left at home due to lack of hospital beds. A massacre. I cry when I think of the call to a lady, just 50 years old: ‘I am at home, they gave me oxygen, I ran out of oxygen [. . .] I struggle to breathe, I do not want to go to the emergency department, I am afraid, I am afraid to die because they have no beds.’" (67)*  *“Knocking at the door, a gentleman with a glass eye and an Elizabethan collar tells me that he has pain in his neck; he should have called me first, but he, like Einstein's hornet, did not know it and came anyway, even without a mask. I did not visit him, I could not, I gathered a good medical history and prescribed the therapy. He thanked me profusely and apologized for coming in without even a shred of protection. And it made me feel like shit. Because I could not help a good and kind person in the best possible way.” (85)*  *"A patient of mine, blind: [she was in] close contact with her manager [. . .] who was COVID-19+ and hospitalized with pneumonia; she was told to quarantine at where she lives with her sister who is immunocompromised and with her elderly father, a real isolation was impossible. No swabs [were performed] because the patient had mild symptoms. Moral of the story, the father is now hospitalized close to death, her sister is hospitalized for COVID-19 pneumonia and she was swabbed only after we threatened [the public health officials] ("we will bring everything to the press")" (95)*    *"In my office, we have only one telephone line where patients can call (for a city of 150,000 inhabitants). It continuously rings." (106)*    *“Unfortunately, the Public Health services are very slow because they have not enough staff and they do not have technologic devices worthy of the name [. . .]. Surely, all surveillance is done on paper.” (107)*    *"To have a swab at home you can turn on a candle. I have been awaiting more than 10 days for its execution, not to speak about the outcome. How do they think they can isolate contacts in this way?"(111)*  *"I regret not being able to give my full contribution due to the lack of adequate PPE." (115)* |
| **Lack of primary care guidelines and protocols** | *"The different ideas about work management, even more when clear guidelines were lacking and in such a delicate phase for everyone, are making teamwork unsustainable. [. . .] I believe that when working in a group we need coordination (even more so at such an exceptional time…) [coordination] is the result of both clear directives given by the institutions and of internal management, aimed to achieve homogeneous conducts [. . .]. I believe that some of the weaknesses of the system have been not to focus on primary care from the beginning, not to create clear outpatient protocols, not to promote greater integration of services, to leave local professionals in uncertainty and to leave patients at home in abandonment." (8)*    *“The delay in updating the protocols: while the epidemic was raging in neighboring territories, according to the regional primary care department protocol we had to ask if the person came from China or from the small number of the first Italian municipalities affected; this regulated "downward" the number of reported cases and the use of PPE. [. . .] My impression was that primary care was left without a leader, without a single authoritative voice from a scientific viewpoint.” (20)*  *"Clear and precise organizational protocols on the management of suspected cases (who to contact in front of a suspected case, possibly by telephone and not by email without having news or answers in a short time); it should be clear and beyond any doubt who should visit the suspected patient and who is responsible for taking him to the emergency department: it is not possible that the OoH doctor calls 112 [emergency number] (according to protocol), but then he is told to go without PPE (or with only a part of them) to manage the patient." (71)*    *"I would try strengthening the primary care service, outlining guidelines so that we know what to do." (76)*  *“During the early stages of the epidemic, when the cases were concentrated mainly in the province of [OMISSIS] but began to multiply in my region [OMISSIS], I had several OoH shifts in a terribly busy ambulatory, especially on Saturdays and Sundays. Despite the concerns expressed by many colleagues about visiting patients provided only with surgical masks and gloves, we have received NO indication about the organization of access to the OoH practice and the precautions to be taken. Throughout the first week of the epidemic, our coordinators did not worry about our safety and working conditions. The whole first week of work has passed in the complete self-organization of the service, with billboards and management paths and procedures produced by the doctors themselves during exhausting WhatsApp discussions [. . .]. This situation generated anger, insecurity, frustration and a sense of abandonment. [. . .] Scientific Societies have to be faster in providing tools and indications for those who work on the frontline and do not have time to conduct reviews of the literature to guide their daily work.” (109)* |
| **Digital technologies** | *“I was also surprised by the rapid possibility of making electronic prescriptions accessible to patients directly from the pharmacy.” (17)*  *"A* *positive experience has been a difficult telephone management of two elderly people aged 80+. I brought them a pulse oximeter and I am calling them twice a day. The husband is as deaf as a post and his wife is very anxious. The management miraculously worked. The wife always told me: ‘You want to know how the finger goes’ and then, in the afternoon, she asked me if I had eaten snacks, while she complained that she had to eat the [OMISSIS-supplementary food]^[1]^" (33)*    *"The rediscovered importance of words, of a telephone conversation that becomes an essential connection, and which can concentrate all possible humanity, closeness and help." (39)*    *"March 10, 6:10 p.m. D.A. calls me, healthcare operator in nursing home in the village of [OMISSIS], a Codiv-19 cluster: ‘Doctor, I got a swab, and I am positive.’ Break. ‘How are you? Fever? Cough? Who do you live with?’ Hence, a telephone and Skype relationship started with D. and his family, father and mother, all infected. Every day I called them, I entered their homes, I saw their eyes, I evaluated their breathing. [. . .] Tomorrow they will repeat the swab. I cross my fingers because I have been living with them for these 20 days.” (42)*    *“During home isolation many people (especially if symptomatic) feel the need to have dedicated assistance that can dispel doubts or fears about the situation, their clinical conditions, all detectable on the phone. That is why the health surveillance work I do is well received by patients in most cases.” (55)* |
| **Doctor-patient relationship** | *“I decided to go [to visit her] after speaking on the phone with a lady who was very anxious. The relief of this person (who had not seen anyone for at least a month) in being reassured after being visited. . . She thanked me. Many others [doctors] refused to visit her. This is one of the very few positive experiences of this period to date.” (8)*    *“The only positive aspect that I can find right now is that patients [. . .] have understood and used the service in an appropriate way.” (10)*    *“Another thing that surprised me positively was hearing a patient asking me: ‘Before starting, doctor, First tell me how you are, because right now you are the people who most need to hear this asked. And maybe nobody does.’” (25)*  *“We were many doctors in that outpatient clinic, without masks. One afternoon a call arrived on my cell phone. It was a friend who informed me that a Chinese girl wanted to donate 400 masks to us. I was very impressed by this great gesture of solidarity. We will be forever grateful to her.” (28)*    *"The understanding of my elderly homebound patients when I had to cancel the planned home visits and the collaboration of their families in helping them and providing medicines for them." (29)*    *“The sense of frustration, loneliness, and uncertainty is becoming very heavy and we share it day by day with our patients. The work of containing our personal anxiety and that of our colleagues and patients are enormous; there are days when this is not possible, and anxiety overwhelms us.” (125)* |
| **Professional collaboration and teamwork** | *Another consideration concerns colleague: I have discovered (or perhaps was confirmed) that some of them may be your strength, your constant mirror, your vent valve, the eyes that most offer you an understanding in such an emotionally and professionally heavy period.” (8)*    *“A colleague from the public health department initially did not want to prescribe the swab I asked for, but then she trusted me, [she believed] that I knew the patient, and that I could know that the reported symptoms were serious. He [the patient] was COVID-19+.” (15)*    *“I was pleased, although not really surprised, with the spirit of collaboration I perceived and I am still perceiving. The feeling of gathering together to cope with a complex situation was quite strong. I observe mutual help (an attitude that was not always previously present). Despite the forced distance, we did not feel alone, or at least, it is true for me. [. . .] To be brief, I think it would be essential to work in multidisciplinary teams (GPs represent only some of the skills needed to provide primary care to the population).” (18)*    *“If I only could have had the help of the nurse to go with PPE to see the elderly homebound COVID-19 patient.” (22)*    *“When I needed to send a patient of mine to the emergency department (ED) for a differential diagnosis between suspected pneumonia or heart failure, I immediately got in touch with my colleagues in the nearby ED and my radiologist colleague and I agreed on the most appropriate and fastest path for my patient. After a few hours, he was out of the ED with the diagnosis made and the therapy set. . . and now he is fine! We rediscovered that sometimes picking up a phone and engaging in human relationships, not just talking to each other through reports, improves performance and helps [us] to work well and feel more satisfied.” (25)*    *"[A positive experience has been] the post-discharge management of a patient, I have been caring for since 19 of March. I collaborated with the cardiologist and with the ADI [integrated homecare service]" (53)*    *“During this epidemic, I found the pleasure of working as a team. I am running a community hospital with a geriatrician and together we discuss cases and think about the management of therapies. I thought about how important it is, also in primary care, to share one's experiences with colleagues and to be able to exchange opinions and thoughts with each other.” (64)*    *“The need to talk to colleagues more often, every day, even several times a day to share what is happening [. . .] the feeling of being alone amplifies distances. Colleagues, at this moment, save you more than anyone else.” (114)*    *"The collaboration started with colleagues in the area where I work. As soon as the first patient in Italy was tested positive, we started communicating on group chats to support each other and exchange information, to get masks, gowns, and oximeters. We adopted a common emergency management line. And, thanks God, we set up the chat, otherwise I would have felt really alone.” (144)* |

[^[1]^](applewebdata://7E482FAC-4805-4255-B6D4-E48F6976226E#_ftnref1) The respondent cited the name of a well-known brand of supplementary food. The brand name was omitted.
